# Differential Changes in Expression of Stress- and Metabolic-Related Neuropeptides in the Rat Hypothalamus during Morphine Dependence and Withdrawal

**DOI:** 10.1371/journal.pone.0067027

**Published:** 2013-06-21

**Authors:** Bernadett Pintér-Kübler, Szilamér Ferenczi, Cristina Núnez, Edina Zelei, Ágnes Polyák, M. Victoria Milanés, Krisztina J. Kovács

**Affiliations:** 1 Laboratory of Molecular Neuroendocrinology, Institute of Experimental Medicine, Budapest, Hungary; 2 Group of Cellular and Molecular Pharmacology, Faculty of Medicine, University of Murcia, Murcia, Spain; University of California, Los Angeles, United States of America

## Abstract

Chronic morphine treatment and naloxone precipitated morphine withdrawal activates stress-related brain circuit and results in significant changes in food intake, body weight gain and energy metabolism. The present study aimed to reveal hypothalamic mechanisms underlying these effects. Adult male rats were made dependent on morphine by subcutaneous implantation of constant release drug pellets. Pair feeding revealed significantly smaller weight loss of morphine treated rats compared to placebo implanted animals whose food consumption was limited to that eaten by morphine implanted pairs. These results suggest reduced energy expenditure of morphine-treated animals. Chronic morphine exposure or pair feeding did not significantly affect hypothalamic expression of selected stress- and metabolic related neuropeptides - corticotropin-releasing hormone (CRH), urocortin 2 (UCN2) and proopiomelanocortin (POMC) compared to placebo implanted and pair fed animals. Naloxone precipitated morphine withdrawal resulted in a dramatic weight loss starting as early as 15–30 min after naloxone injection and increased adrenocorticotrophic hormone, prolactin and corticosterone plasma levels in morphine dependent rats. Using real-time quantitative PCR to monitor the time course of relative expression of neuropeptide mRNAs in the hypothalamus we found elevated CRH and UCN2 mRNA and dramatically reduced POMC expression. Neuropeptide Y (NPY) and arginine vasopressin (AVP) mRNA levels were transiently increased during opiate withdrawal. These data highlight that morphine withdrawal differentially affects expression of stress- and metabolic-related neuropeptides in the rat hypothalamus, while relative mRNA levels of these neuropeptides remain unchanged either in rats chronically treated with morphine or in their pair-fed controls.

## Introduction

Opiate addiction results in significant changes in mood and affective behaviour, while morphine withdrawal syndrome consists of severe somatic alterations [1], whose neurobiological background have not been fully resolved. Understanding activation of the stress-related brain circuit and changes in energy metabolism and feeding behaviour during drug withdrawal is important because these alterations have been reported to be among the most severe physical symptoms of morphine administration and withdrawal. Morphine treatment is often accompanied by robust changes in food intake and body weight. In the first part of this study, we aimed to follow, day-by-day, changes in these metabolic parameters of rats implanted with placebo or morphine pellets. An additional group of placebo implanted rats was pair fed to morphine implanted animals such that the amount of food provided to these animals was equal to that consumed by the morphine-treated group. This pair fed group permitted the investigation of the morphine’s effect on energy balance via mechanisms independent of food intake.

Chronic morphine exposure and withdrawal results in neuronal plasticity in the brain stress system and affects neuroendocrine- and autonomic regulation. Upregulated corticotropin-releasing hormone (CRH) and arginine vasopressin (AVP) transcription in the hypophyseotropic neurosecretory neurons of the hypothalamic paraventricular nucleus (PVN) is related to activation of hypothalamo-pituitary-adrenocortical (HPA) axis during naloxone precipitated morphine withdrawal [2], [3].

Proopiomelanocortin (POMC) and neuropeptide Y (NPY) synthesizing hypothalamic cell groups have been recognized to coordinate food intake and energy metabolism (for recent review see [4]). Morphine withdrawal is accompanied with significant metabolic alterations that might also be governed by these hypothalamic networks. For instance, it is well demonstrated that naloxone precipitated- or spontaneous opioid withdrawal results in weight loss up to 15–20% of body weight in rats [5]. In spite of these significant somatic alterations it remained unknown how abused drugs and drug withdrawal affects metabolic-related neurocircuits. Functional anatomical mapping using immediate-early genes have repeatedly revealed recruitment of neurons in different hypothalamic (arcuate, ventromedial and paraventricular nuclei), limbic (amygdala) and medullary (NTS) structures that are parts of neuronal circuits involved in stress- and metabolic regulation. These immediate early genes encode transcription factors that may regulate neuropeptide expression and the encoded peptides are involved in regulation of food intake and energy expenditure during morphine dependence and withdrawal.

It is well recognized that CRH and related urocortins have important metabolic effects in the CNS and at the periphery and have been implicated in coordination of autonomic functions during stress [6]. Furthermore, urocortins have recently been implicated in neuroadaptations that contribute to development of alcohol and cocaine addiction [7] although their involvement in morphine addiction and withdrawal has not been addressed yet.

We hypothesize that robust stress- and metabolic changes seen in morphine withdrawal are accompanied with transcriptional activity of key neuropeptide genes in the hypothalamus. Thus, in the second part of this study we measured and followed the timing of changes during naloxone-precipitated morphine withdrawal on HPA activity, energy balance as well as on the transcription of stress-related- anorexigenic- and orexigenic neuropeptides within the hypothalamus.

## Materials and Methods

### Animals

Adult male Wistar rats (from the colony breed at the Institute of Experimental Medicine Budapest) weighing 250–300 g at the beginning of the experiments were used. Animals had free access to rodent food and water and were maintained under controlled conditions: temperature, 21°C±1°C; humidity, 65%; light-dark cycle, 12-h light/12-h dark cycle, lights on at 07∶00. All procedures were conducted in accordance with the guidelines set by the European Communities Council (86/609/EEC/2 and 2010/63 Directives of European Community) and the protocol was approved by the Institutional Animal Care and Use Committee of the Institute of Experimental Medicine, Budapest Hungary (permit numbers: 22.1/3347/003/2007 and (PEI/001/29-4/2013).

### Morphine Pellet

Morphine base was obtained from Alcaliber Laboratories (Madrid, Spain) in cooperation with the Área de Estupefacientes y Psicotropos, Agencia Española del Medicamento y de Productos Sanitarios (Madrid, Spain). Pellets of morphine and lactose (control) were prepared in the Department of Pharmacy and Pharmaceutics Technology (School of Pharmacy, Granada, Spain).

### Experimental Procedures

**Figure 1:**
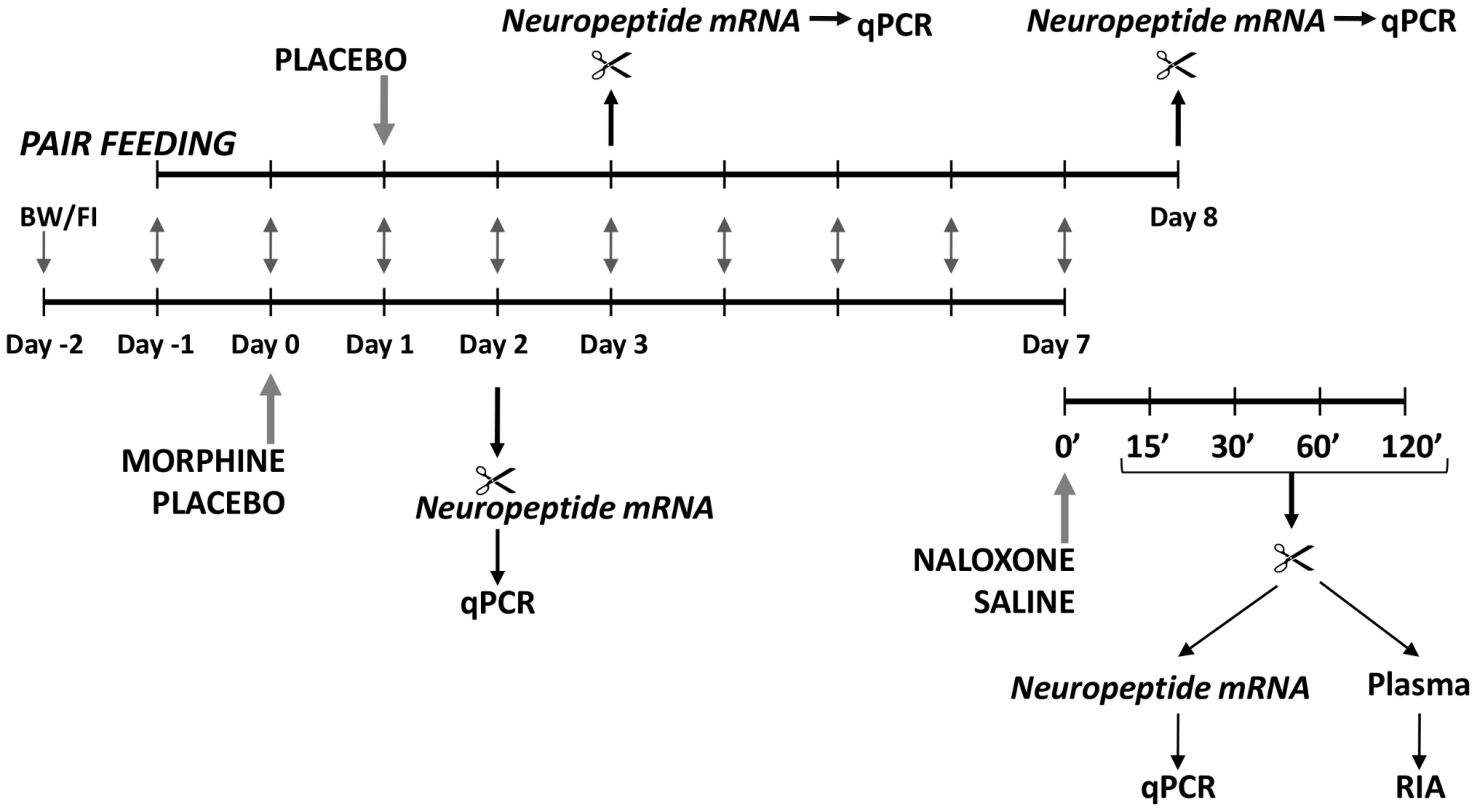
Schematic overview of the pair feeding and time course experiments. Baseline food intake (FI) and body weight (BW) data were obtained on days −2 and −1. On day 0 rats were subcutaneously implanted with placebo or morphine pellets. Based on the food consumption data of morphine implanted rats, a group of placebo implanted, pair fed rats was added on day 1. These rats received the same amount of food daily that was consumed by their morphine-implanted pairs on the previous day. Some morphine and placebo treated rats were decapitated on day 2 along their placebo-implanted, pair fed partners on day 3. On day 7, placebo and morphine-implanted groups were further divided into two groups half of the rats from each group received subcutaneous injection of saline or naloxone and sacrificed at different time points thereafter. Animals from pair fed group were decapitated on day 8 (7^th^ day after pellet implantation).

Flow chart of the experiments is shown on Figure 1.

### Experiment 1. Morphine Treatment and Pair Feeding

Before the experiment, rats were handled and their food consumption and body weight registered daily. Animals were rendered dependent on morphine by subcutaneous implantation of constant release morphine base pellets (2×75 = 150 mg) on day 0, under light ether anaesthesia. Control animals were implanted with placebo pellets containing lactose instead of morphine. Animals were kept in individual cages, their body weight and food consumption was recorded daily (between 08–09 am). On day 2 and day 7, animals were decapitated, trunk blood collected, hypothalami were dissected and frozen for qRT-PCR measurements.

One day after pellet implantation (day 1), a third group of rats, matched for body weight to the morphine treated group, was implanted with placebo pellet (pair fed group). Animals in this group received the same amount of food consumed by their morphine implanted pairs in the previous day. This group of animals was sacrificed on day 3 or day 8.

### Experiment 2. Naloxone Precipitated Morphine Withdrawal

Separate set of rats were implanted with morphine or placebo pellets as described above. In the morning of day 7, animals were weighted and placed into transparent plastic cages in a quiet room and left undisturbed for 15 min. Rats were then injected subcutaneously with naloxone (Sigma, 1 mg/kg/ml bw) or saline (1 ml/kg bw) and decapitated 2 hours after injection.

Two independent observers, unaware of the drug combination used, observed the rats for the occurrence of somatic signs of opiate withdrawal up to 15 min after the naloxone or saline injections. The following behavioural elements were recorded onto a preformulated score sheet: somatic signs: jumps, wet-dog shakes, paw tremors, sniffing, ptosis, mastication, and body tremor [8], and vegetative signs: diarrhoea, rhynorrhea, lacrimation and piloerection [9].The withdrawal-induced weight loss of the animals was calculated as the difference between body weights before naloxone/saline injection and before decapitation.

Trunk blood was collected into ice cold EDTA containing plastic tubes, centrifuged and plasma samples for hormone measurement (adrenocorticotrophic hormone, (ACTH), corticosterone, insulin, adiponectin and leptin) were stored −20°C. After decapitation, a drop of blood was applied to the D-Cont Personal blood glucose meter (77 Elektronika LTD Budapest, Hungary) for blood glucose measurement.

Hypothalamic tissue blocks for real-time PCR were also collected and stored at −70°C.

### Experiment 3. Time Course of Naloxone Precipitated Morphine Withdrawal

To reveal time-dependent changes of HPA axis activation and in neuropeptide expression during morphine withdrawal, separate set of rats were made morphine-dependent on day 0. The food consumption and body weight was also recorded daily. On day 7, animals were weighted and injected subcutaneously with naloxone (Sigma; 1 mg/kg; 1 ml/kg bw) then were decapitated at various time intervals (15, 30, 60, 120 min) after injection. Placebo-implanted control animals received the same volume (1 ml/kg) saline subcutaneously.

The behaviour of the animals was observed during the first 15 min of withdrawal as in experiment 2. The weight loss was also calculated as a difference between pre – and post injection values. Blood samples for ACTH, corticosterone and prolactin (PRL) were collected from trunk blood. Hypothalamic samples were dissected and stored as in experiment 2.

### Hormone Measurements

Plasma adrenocorticotrophic hormone (ACTH), prolactin (PRL) and corticosterone (CORT) concentrations were measured by radioimmunoassay (RIA) as described. ACTH RIA was developed in our group [10] using an antibody (#8514) raised against the mid-portion of human ACTH1-39. The test uses 50 µl of plasma per determination, has a lower limit of sensitivity of 0.1 fmol/ml, and the average intra-and inter-assay coefficients of variation are 4.8% and 7.0% respectively. Plasma corticosterone has been measured by a direct RIA without extraction as described [11]. The intra and interassay CVs in this assay are 12.3 and 15.3 respectively.

Because plasma prolactin (PRL) in males is a relevant indicator of stress and PRL release is under dopaminergic control by tuberoinfundibular dopaminergic (TIDA) neurons, we followed the time course of PRL secretion during withdrawal by measuring plasma hormone levels by RIA. The sensitivity of PRL assay is 2 ng/ml, with 10.53% intra- and 12.67% interassay variation.

Plasma concentrations of leptin, insulin and adiponectin were measured by RIA kits obtained from Linco/Millipore according to the manufacturer’s instructions.

### Hypothalamic Neuropeptide mRNA Levels

After decapitation, the brain was immediately removed from the skull, placed onto RNase free rubber surface and the hypothalamus was dissected with a sterile razor blade. The boundaries of the hypothalamic blocks were at the optic chiasm in rostral- at the mammillary bodies in caudal- and at the hypothalamic sulcus in the lateral directions. The tissue samples were immediately frozen on dry ice and stored at −70°C until assay.

### Primer Design

Primers used for the comparative C_T_ experiments were designed by the Primer Express 3.0 program (Applied Biosystems). Primer sequences are given in Table 1.

**Table 1:** List of primer pairs used in real-time quantitative PCR reactions.

**GAPDH**
Forward: ACAGCCGCATCTTCTTGTGC
Reverse: GCCTCACCCCATTTGATGTT
**CRH**
Forward: CAGCCGTTGAATTTCTTGCA
Reverse: CCAGGCGGAGGAAGTATTCTT
**UCN2**
Forward: GGATGTCCCCATTGGCCTCCTG
Reverse: GCGGCCAACACGGGCTAGTA
**POMC**
Forward: AGGTTAAGGAGCAGTGACTAAG
Reverse: CGTCTATGGAGGTCTGAAGC
**NPY**
Forward: CCATGATGCTAGGTAACAAACGAATG
Reverse: ATGTAGTGTCGCAGAGCGGAGTA
**AVP**
Forward: TCGCCATGATGCTCAACACT
Reverse: CTCTTGGGCAGTTCTGGAAGTAG

### Quantitative Real-Time PCR

Total RNA was isolated from hypothalamic samples with QIAGEN RNeasy Mini Kit (Qiagen, Valencia, CA, USA) according the manufacturer’s instruction. To eliminate genomic DNA contamination DNase I treatment was used (100 µl RNase-free DNase I (1 U DNase I, Fermentas) solution was added). Sample quality control and the quantitative analysis were carried out by NanoDrop (Thermo Scientific). Amplification was not detected in the reverse transcription (RT)-minus controls. cDNA synthesis was performed with the High Capacity cDNA Reverse Transcription Kit (Applied Biosystems, Foster City, CA, USA). The designed primers (Invitrogen) were used in the Real-Time PCR reaction with Power SYBR Green PCR master mix (Applied Biosystems, Foster City, CA, USA) on ABI StepOne instrument. The gene expression was analyzed by ABI StepOne 2.0 program. The amplicon was tested by Melt Curve Analysis on ABI StepOne instrument. GAPDH was used as endogenous control reference genes and all data were normalized to GAPDH expression.

### Statistical Analysis

Data are presented as mean ± SEM. Body weight, food intake (Experiment 1) and time xcourse of hormone levels and neuropeptide mRNA during withdrawal (Experiment 3) were analysed by one-way analysis of variance (ANOVA) with time (days and minutes, respectively) as the repeated measure and treatment as the main factor using Prism6.1 software (GraphPad, La Jolla, CA). One-way ANOVA with Tukey’s HSD test was used to assess the effect of treatment at each particular time point (2 or 7 days or 0, 15, 30, 60, 120 min). Body weight loss, plasma hormone and glucose levels as well as neuropeptide mRNA levels in Experiment 2 (two hours withdrawal) were analysed by two-way ANOVA with chronic treatment (morphine/placebo pellet) and acute injections (naloxone/saline) as main factors. To compare two groups, Student's *t-* test was used. In all cases *p* value of 0.05 or lower was considered significant.

## Results

### Experiment 1. Chronic Morphine Treatment and Pair Feeding

#### Body weight, food and water intake

**Figure 2:**
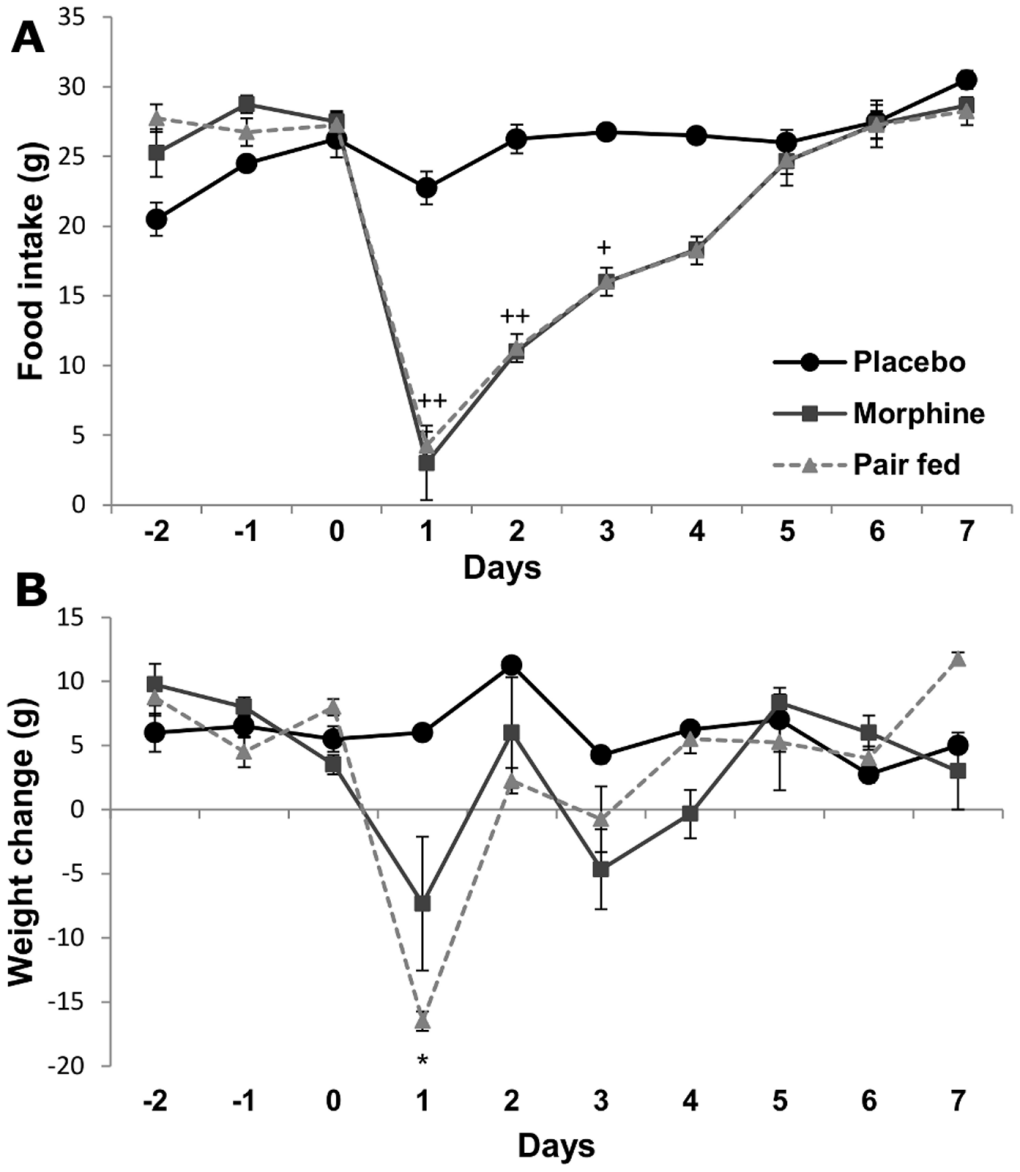
Effects of chronic morphine treatment and pair feeding on metabolic parameters of male rats. Daily changes of food intake in placebo and morphine implanted animals. Pair fed rats were placebo implanted and received the same amount of food that was consumed by the morphine implanted pairs. n = 12, ^+^ *p*<0.05, ^++^ *p*<0.01 morphine vs. placebo-implanted group (Student’s t-test) A. Mean (±SEM) values of daily changes in body weight of morphine implanted and placebo implanted and pair fed rats. Note that pair feeding of placebo implanted rats resulted in significantly more body weight loss compared to morphine implanted animals, n = 12, *p<0.05 placebo implanted vs. pair fed group (Student’s t-test).

Following morphine implantation (day 1 post surgery) animals consumed 87% less food compared to placebo implanted controls (Fig. 2A). This decrease of food intake was accompanied by a significant reduction in body weight gain (Δ bw24 h after placebo: +6.0±0.5 g, morphine: −7.3±2.5 g). Tolerance developed in food intake after day 2 post implantation in morphine implanted rats. Placebo-implanted pair fed rats lost significantly more weight during the first three days after pellet implantation than their morphine implanted pairs (Fig. 2B).

Chronic morphine administration decreased cumulative water intake over the 7 days experimental period (placebo: 320.3±39.0 ml vs. morphine 184.2±17.5 ml, p = 0.019 Student’s t-test).

#### Plasma hormone levels

Table 2. summarizes changes in plasma ACTH, CORT and PRL levels in placebo, morphine implanted- and pair fed animals. ANOVA on CORT data revealed significant treatment effect F_(2,15)_ = 6.79, p = 0.0095. Tukey’s post-hoc test indicated significantly (p<0.05) elevated CORT levels in morphine implanted rats compared either to placebo or pair fed animals two days after pellet implantation.

**Table 2:** Mean±SEM plasma hormone concentrations in placebo- or morphine implanted and pair-fed rats 2 or 7 days after pellet implantation.

	2 days			7 days		
	Placebo	Morphine	Pair Fed	Placebo	Morphine	Pair Fed
**ACTH (pM)**	8,05±4,02	22,35±11,52	20,80±5,16	49,20±18,43	34,87±3,57	59,05±15,76
**CORT (nM)**	128,1±94,1	1365,4±443,7* ^+^	282,5±129,3	515,2±365,1	1622,5±937,4	1682,5±325,6
**PRL (ng/ml)**	6,06±2,38	4,10±1,58	6,60±2,73	5,32±1,33	7,85±3,98	17,57±3,90

* *p*<0,05 morphine vs. pair fed;^ +^ *p*<0,05 placebo vs. morphine; n = 7/group.

#### Neuropeptide mRNA

Relative mRNA levels of arginine vasopressin AVP, corticotropin-releasing hormone CRH, proopiomelanocortin POMC and urocortin 2 (UCN2, also known as stresscopin-related peptide in human) were not significantly different in hypothalamic blocks of morphine treated animals, placebo implanted-, and pair fed rats that were sacrificed 2 or 7 days after treatment (Table 3). In case of NPY, ANOVA showed significant treatment effect in groups of animals 2 days (F_(1,21)_ = 20.51, p<0.001) and 7 days (F_(1,21)_ = 12.85 p = 0.032) after pellet implantation. Elevated hypothalamic NPY mRNA level was detected 2 days after morphine implantation compared to placebo implanted control and pair fed groups. Pair fed rats displayed elevated NPY mRNA levels seven days after placebo pellet implantation (Table 3).

**Table 3:** Mean±SEM relative expression levels of selected hypothalamic neuropeptides in placebo or morphine-treated and pair fed animals 2 or 7 days after pellet implantation.

	2 days			7 days		
	Placebo	Morphine	Pair Fed	Placebo	Morphine	Pair Fed
**CRH**	1,00±0,15	0,95±0,10	1,10±0,06	1,00±0,14	0,97±0,31	1,14±0,18
**UCN2**	1,00±0,23	1,51±0,36	0,71±0,04	1,00±0,28	0,83±0,30	0,92±0,07
**NPY**	1,00±0,06	1,55±0,07^+^	1,22±0,09^#^	1,00±0,09	0,92±0,11	1,55±0,09* ^#^
**POMC**	1,00±0,15	1,14±0,13	0,82±0,09	1,00±0,08	0,67±0,17	0,86±0,15
**AVP**	1,00±0,16	1,17±0,18	0,98±0,15	1,00±0,18	1,10±0,16	1,26±0,10

* *p*<0,01 placebo vs. pair fed; ^#^ *p*<0,01 morphine vs. pair fed; ^+^ *p*<0,001 placebo vs. morphine; n = 7/group.

### Experiment 2. Morphine Withdrawal

In this experiment the following groups were tested: placebo+saline, placebo+naloxone, morphine+saline and morphine+naloxone. All animals were implanted with subcutaneous pellets for 7 days and decapitated 2 hours after saline/naloxone injection. Categories for statistical analysis were: placebo or morphine pellet implantation-(chronic) treatment; saline or naloxone injection-(withdrawal).

#### Behaviour

Chronic morphine exposure produced strong physical dependence syndrome as assessed by the characteristic set of behavioural responses to naloxone-evoked withdrawal [12]. All (100%) morphine implanted, naloxone injected rats displayed tremor, ptosis and escape; 80% showed salivation, rhynorrhea, teeth chattering and “wet-dog” shakes; while lacrimation was observed in 77%. 60% of rats displayed diarrhoea and 55% showed piloerection during the 15 min observation period. None of these behavioural elements was observed in the other treatment groups.

#### Weight loss

Naloxone precipitated morphine withdrawal resulted in significant weight loss (−26.8±1.9 g) in 2 hours after naloxone injection (F_(3,14)_ = 59.2, p<0.001). The weight change in any other treatment group was not significant (placebo+saline: −1,9±0,5 g; placebo+naloxone: −24±0.3 g; morphine+saline: −1.67±0.2 g).

#### Hormones

As shown in Table 4, plasma ACTH and CORT levels were elevated in morphine implanted rats two hours after naloxone injection compared to placebo implanted control animals [ACTH: F_(1,39)_ = 24.69, p<0.001 treatment and F_(1,39)_ = 18.40, p<0.001 withdrawal; CORT: F_(1,39)_ = 62.12, p<0.001 treatment and F_(1,39)_ = 65.81, p<0.001 withdrawal]. Morphine dependent, saline injected or placebo implanted, naloxone injected rats did not display significantly altered plasma concentration of ACTH or CORT. Plasma PRL concentrations were not different in morphine dependent or placebo implanted animals sacrificed two hours after saline/naloxone treatment. Table 4. also summarises changes in blood glucose and plasma insulin, leptin and adiponectin levels as measured at 2 hours after saline or naloxone injection.

**Table 4:** Plasma hormone levels and blood glucose concentration 7 days after morphine or placebo implantation and 2 hours after saline or naloxone injection.

	Placebo		Morphine	
	Saline	Naloxone	Saline	Naloxone
**ACTH (pM)**	55,93±11,20	29,96±9,71	58,96±16,24	451,53±73,53*
**CORT (nM)**	493,74±87,85	306,85±72,88	249,26±54,86	4479,3±378,5*
**PRL (ng/ml)**	34,26±7,01	31,96±13,91	25,35±6,51	35,40±13,27
**Blood Glucose (mM)**	8,68±0,84	7,25±0,25	6,75±0,56	11,03±0,61*
**Insulin (ng/ml)**	1,93±0,15	1,82±0,43	1,15±0,42	1,34±0,04
**Leptin (ng/ml)**	2,08±0,26	2,02±0,18	0,85±0,11+	1,82±0,06*
**Adiponectin (µg/ml)**	2,26±1,53	4,08±0,86+	2,46±0,56	2,14±0,54

+ p<0,05 placebo+saline vs. placebo+naloxone; *p<0,05 morphine+saline vs. morphine+naloxone group; n = 6/group.

Chronic morphine treatment had no effect on blood glucose concentration compared to placebo implanted controls, however elevated blood glucose levels were detected in the group of morphine implanted, naloxone treated rats.

In *ad libitum* fed animals, leptin plasma levels were not different in naloxone injected groups compared to placebo-implanted saline injected controls. However, a moderate decrease in plasma leptin concentration was detected after chronic morphine treatment, which has increased 2 h after naloxone injection.

Plasma adiponectin level was significantly higher in placebo implanted, naloxone injected rats compared to any other group.

Plasma insulin levels did not show any significant difference in response to chronic morphine administration or withdrawal.

#### Neuropeptide mRNA in the hypothalamus

**Figure 3:**
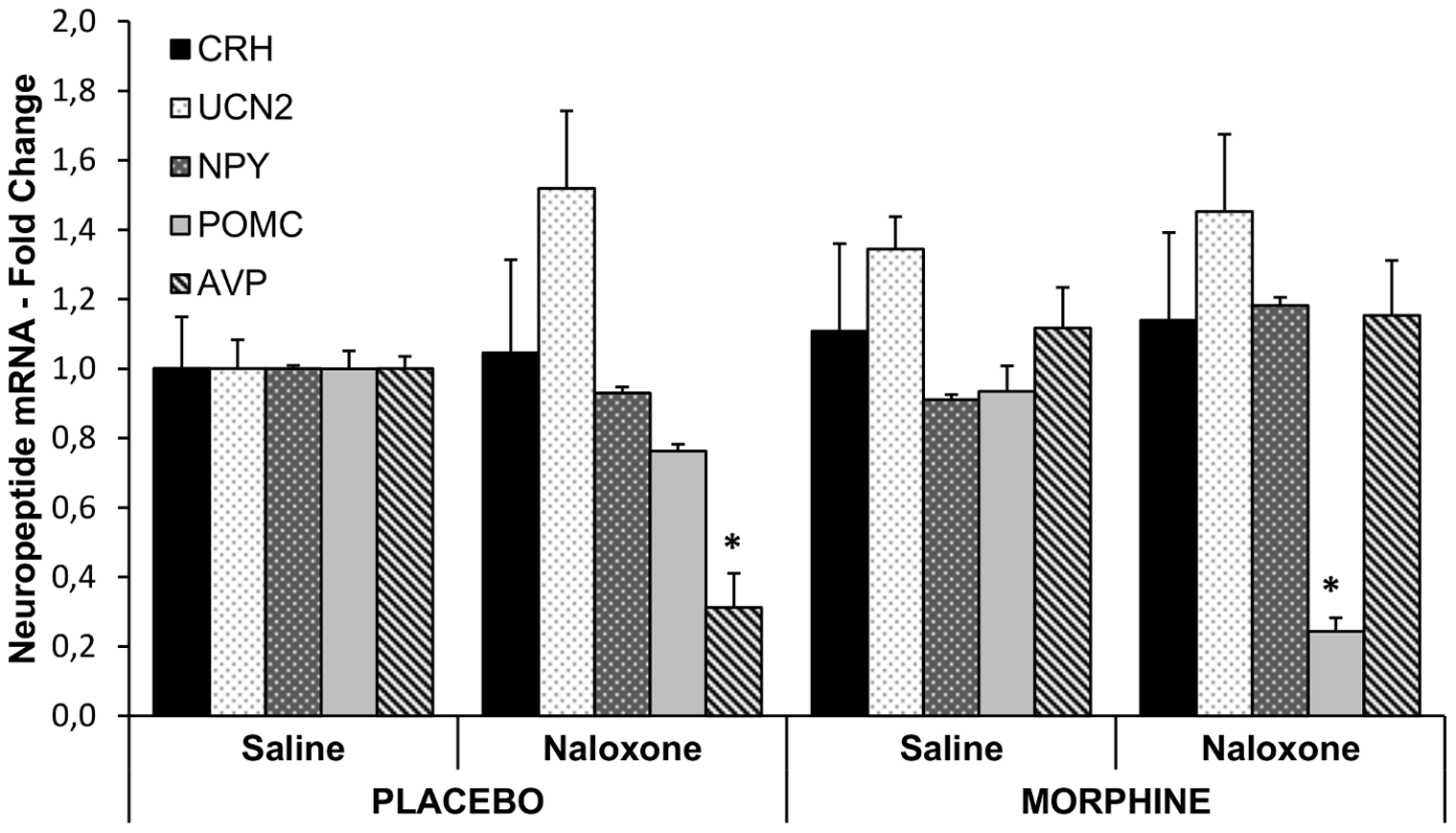
Effect of chronic morphine treatment and naloxone precipitated morphine withdrawal on neuropeptide expression in the hypothalamus. Mean (±SEM) values of relative expression values of selected hypothalamic neuropeptide mRNAs in placebo or morphine treated rats two hours after saline or naloxone injection. Neuropeptide mRNA levels were normalised to GAPDH levels and expressed as fold change compared to the levels of placebo implanted saline groups set to 1. n = 6/group, *p<0.0.1 compared to saline injected respective controls.

Morphine dependence and two hours’ opiate withdrawal affected AVP and POMC mRNA levels in the hypothalamus. Relative quantities of AVP mRNA were significantly lower in placebo implanted naloxone injected rats than in placebo implanted saline injected animals and in both morphine treated groups (chronic treatment, F_(1,10)_ = 18.76, p = 0.0015; acute treatment F_(1,10)_ = 8.55, p = 0.015; chronic×acute treatment interaction, F_(1,10)_ = 11.04, p = 0.007). Morphine withdrawal resulted in a significant decrease of hypothalamic POMC mRNA levels two hours after naloxone injection (chronic treatment, F_(1,20)_ = 2.368, p = 0.13 NS; acute treatment, F_(1,20)_ = 17.61, p<0.001; chronic treatment×acute treatment, F_(1,20)_ = 0.11, p = 0.74 NS). RQ values for CRH, UCN2 and NPY were not significantly different in the hypothalamus of morphine dependent rats two hours after morphine withdrawal (Fig. 3).

### Experiment 3. Time course of Changes During Naloxone-Precipitated Morphine Withdrawal

In this set of experiments the time course of weight loss, hormonal- and neuropeptide responses were followed during naloxone precipitated morphine withdrawal and compared to that of placebo implanted, saline injected animals.

Behaviour of the rats during withdrawal that was observed during the first 15 min following saline/naloxone injections was similar to that observed in Experiment 2.

#### Time course of withdrawal-induced weight loss

Two way ANOVA revealed significant effect of treatment, F_(1,29)_ = 198.4, p<0.001; time, F_(3,29)_ = 15.21, p<0.001 and treatment×time interaction, F_(3,29)_ = 11.91 p<0.001.

**Figure 4:**
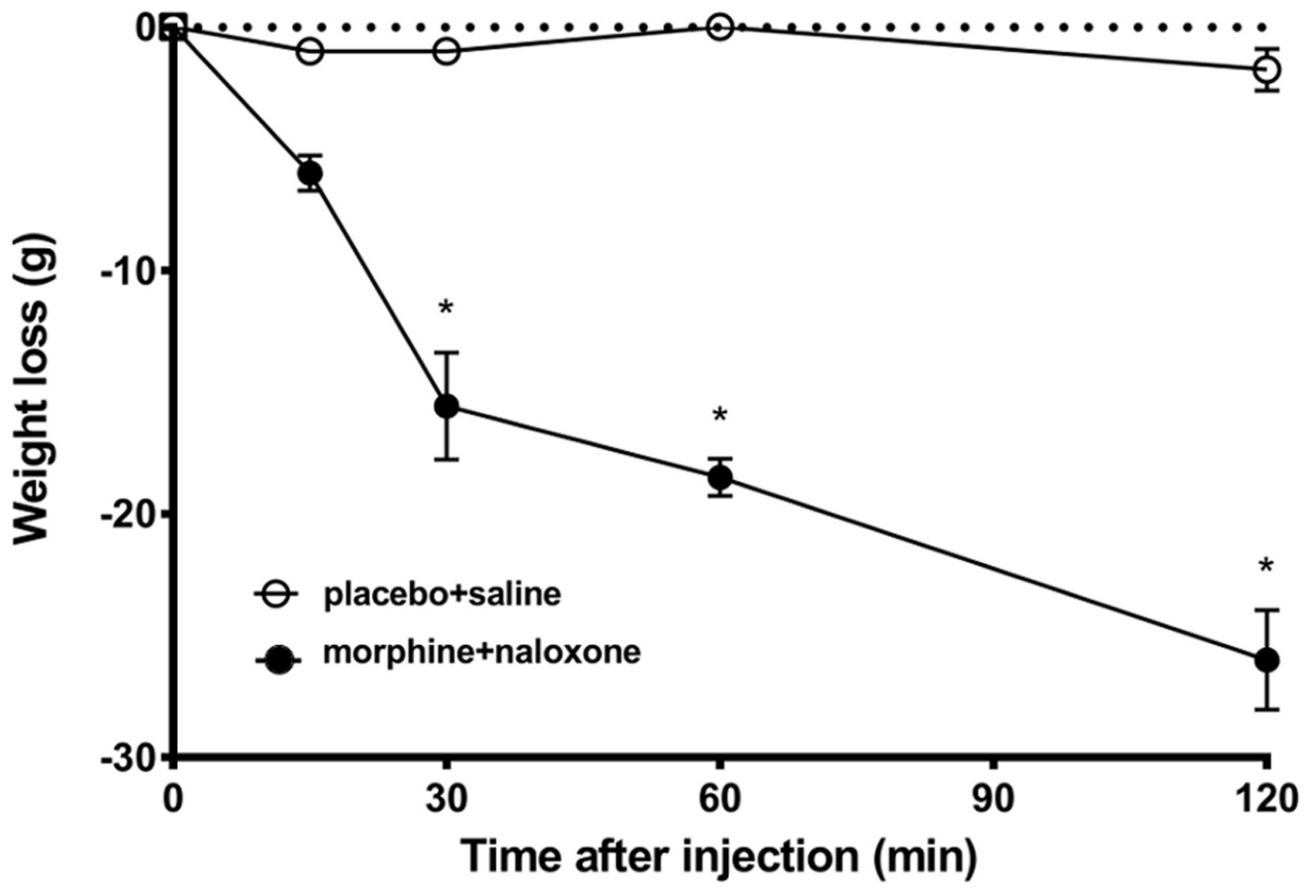
Time course of weight loss during morphine withdrawal. Mean (±SEM) values of body weight change in morphine implanted, naloxone injected- (solid dots) and placebo implanted, saline injected rats (open dots) at different time points after injection. n = 16/group, p<0.01 compared to time 0.

Figure 4. shows that body weight gradually decreases during morphine withdrawal and the weight loss is significant as early as 30 min after naloxone injection to morphine dependent rats. Saline injection stress in placebo implanted animals did not result in any significant body weight change during the two hours experimental period.

#### Time course of HPA axis activation and prolactin secretion

**Figure 5:**
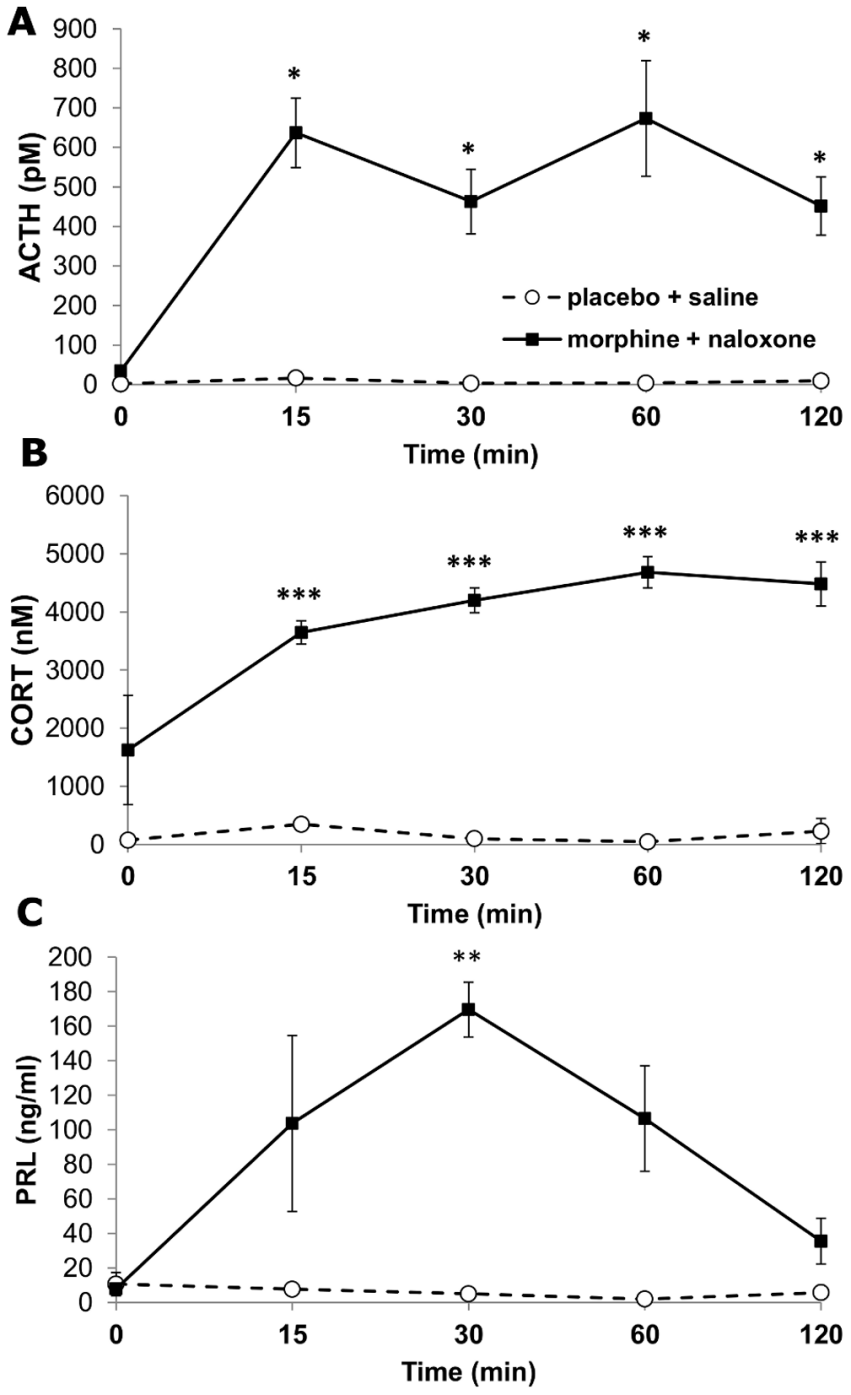
Changes in plasma hormone levels during morphine withdrawal. Mean (±SEM) values for adrenocorticotrophic hormone ACTH (A) corticosterone (B) and prolactin, PRL (C) plasma levels in morphine implanted naloxone injected- and placebo treated saline injected rats. n = 16/group; *p<0.05; **p<0.01; ***p<0.001.

ANOVA revealed significant treatment effect on plasma ACTH level during morphine withdrawal (treatment F_(1,23)_ = 27.94, p<0.001; time, F_(4,23)_ = 1.57 p = 0.23 NS; interaction, F_(4,23)_ = 1.63, p = 0.19 NS). Naloxone precipitated morphine withdrawal dramatically enhanced ACTH secretion, compared to saline injection, as early as 15 min after challenge, remained elevated up to 2 hours after naloxone injection. (Fig. 5A). ACTH levels were not significantly different in placebo implanted, saline injected rats.

As shown in Fig. 5B, plasma CORT levels were significantly elevated as early as 15 min after naloxone injection in morphine treated animals and remained at this plateau level during the whole 120 min testing period. In placebo implanted rats saline injection did not result in any significant change of CORT plasma levels. (ANOVA results: treatment, F_(1,34)_ = 1789, p<0.001; time, F_(4,34)_ = 127, p<0.001.).

Changes in plasma PRL concentration following morphine withdrawal were significant (treatment, F_(1,23)_ = 119.98, p<0.001; time, F_(4,23)_ = 14.5, p<0.001). While PRL levels were not different in placebo implanted saline injected rats, naloxone administration of morphine implanted rats resulted in an elevation of PRL secretion at 15 min, with a significant peak at 30 min and declined by 2 hours post-injection (Fig. 5C).

#### Time course of hypothalamic neuropeptide expression

**Figure 6:**
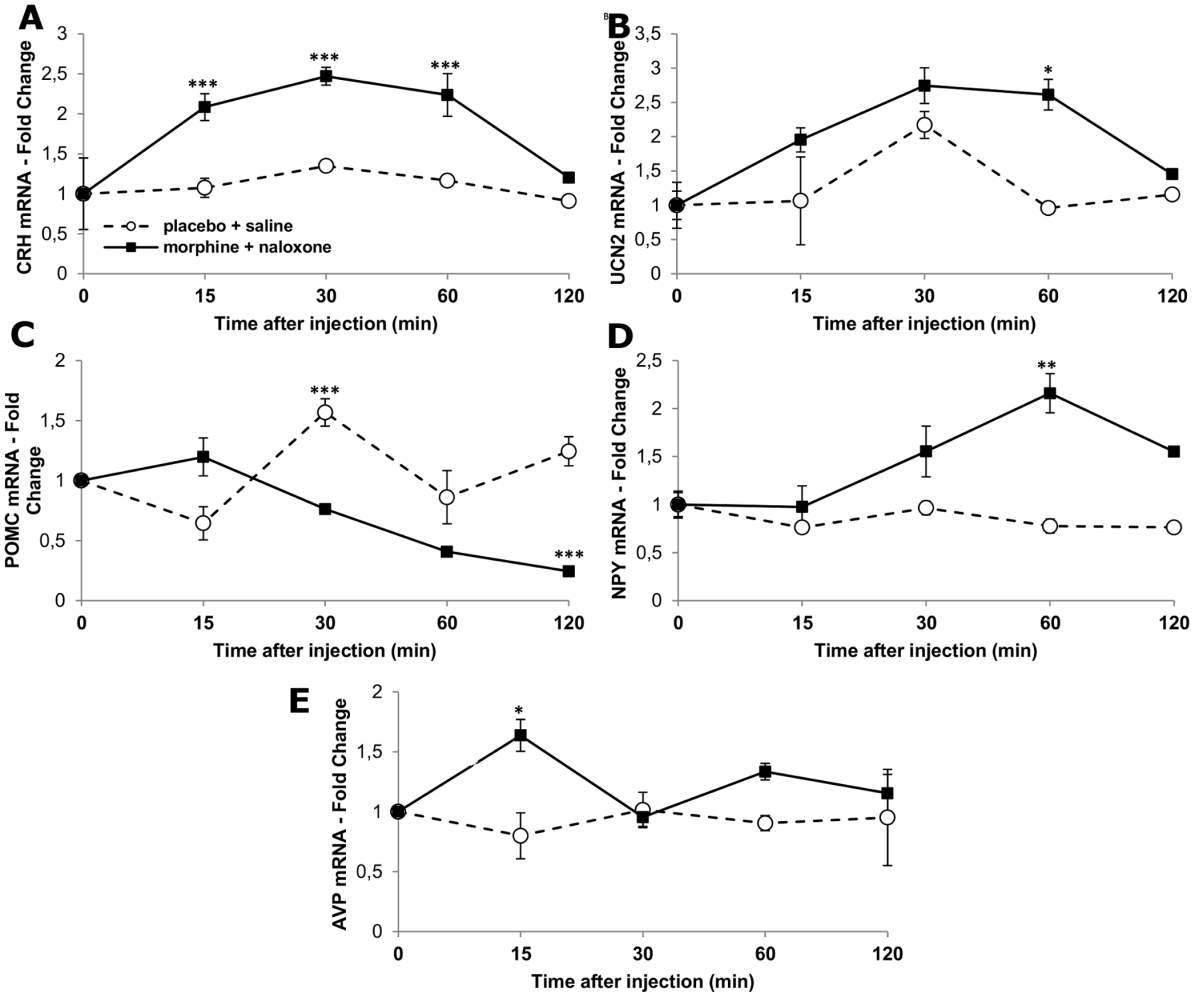
Time course of relative expression levels of stress- and metabolic-related neuropeptide genes in the hypothalamus during naloxone precipitated morphine withdrawal. Mean (±SEM) fold change values of CRH, UCN2, POMC, NPY and AVP mRNAs in the hypothalamus of morphine-implanted naloxone injected (solid bars) compared to placebo-implanted saline injected animals (open bars). All values were normalized to GAPDH levels and shown as fold changes compared to levels of morphine and placebo implanted animals sacrificed at time 0, respectively. n = 5/time point, *p<0.05; **p<0.01; ***p<0.001, compared to placebo implanted, saline injected group (at the same time point).

Figure 6. shows the time course of relative fold changes of corticotropin-releasing hormone (CRH), urocortin 2 (UCN2), proopiomelanocortin (POMC), neuropeptide Y (NPY) and arginine vasopressin (AVP) mRNAs in the hypothalamic samples of morphine dependent rats following withdrawal as compared to placebo implanted, saline injected animals. Naloxone precipitated morphine withdrawal resulted in significant increase of CRH (treatment, F_(1,24)_ = 129.60, p<0.001; time, F_(4,24)_ = 33.5, p<0.001), UCN2 (treatment, F_(1,24)_ = 54.41, p<0.001; time, F_(4,24)_ = 19.93, p<0.001), NPY (treatment, F_(1,24)_ = 206.60, p<0.001; time, F_(4,24)_ = 15.45, p<0.001); and AVP (treatment, F_(1,24)_ = 18.7, p<0.001; time, F_(4,24)_ = 1.23, p = 0.32 NS) mRNA relative quantities in the hypothalamus. Expression of POMC mRNA was continuously decreased in the hypothalamus following naloxone precipitated morphine withdrawal to 30% of the baseline by 120 min after naloxone injection (treatment, F_(1,24)_ = 71.99, p<0.001; time, F_(4,24)_ = 19.45, p<0.001).

## Discussion

### Methodical Consideration

There are two distinct experimental protocols with which to address the effects of chronic opioid exposure in rodents. Repeated injections of escalating doses of morphine result in cycles of partial morphine withdrawal and mimic the situation of addicts using short acting opioids. By contrast, subcutaneous implantation of slow release morphine pellets has been repeatedly shown to maintain constant level of morphine in rats to induce tolerance and physical dependence [8], [13]. This paradigm is similar to the situations when patients are on chronic morphine therapy or when addicts are given long acting opioid methadone during detoxification [14]. In this study we have implanted 2 morphine pellets at the beginning of the experiments, instead of implanting increasing amounts of morphine pellets in every second day as reported in previous protocols [15], [16] to avoid the effect of surgical stress. Subcutaneous implantation surgery in ether anaesthesia is an important stress factor that affects food intake, body weight, HPA activity and related neuropeptide expression [17]. Our present drug treatment paradigm has also been shown to produce constant plasma level of morphine throughout the experiment and a full withdrawal syndrome following acute injection of opiate antagonists [8] . Here we also found somatic and behavioural withdrawal syndrome in morphine implanted rats following naloxone injection.

### Morphine Dependence

Similar to other morphine treatment regimes [16], [18], [19] subcutaneous implantation of 150 mg morphine also resulted in a dramatic reduction in food consumption and body weight gain, which was significant in the first two days after drug administration. To investigate if body weight loss is due to decrease in food intake and/or changes in energy metabolism pair-feeding experiments were performed. Here we report that weight loss of morphine implanted animals, is less than that seen in placebo-implanted pair fed rats, suggesting decrease in their metabolic activity. Indeed, it has been well documented that morphine reduces gut motility and energy metabolism [20] which may contribute to the less weight loss seen in morphine–treated animals compared to pair fed animals. Furthermore, hypo-locomotion seen in morphine-implanted rats may also contribute to reduced weight loss [21].

We have found dissociation of HPA axis regulation in the early phase of chronic morphine administration. Plasma CORT levels were significantly elevated in morphine treated rats 2 days following pellet implantation without any increase of hypothalamic CRH mRNA or plasma ACTH. Increased CORT secretion seems to be directly related to morphine administration rather than a consequence of surgical stress or decreased food intake and weight loss, since placebo implanted and pair fed rats did not have increased CORT plasma concentration.

HPA axis activity is clearly different in the early (2 days) and late (7 days) phase of chronic morphine implantation. By seven days post-implantation there is no significant difference in ACTH and CORT levels of morphine dependent and placebo treated animals and -in agreement with previous reports [3], [22]-we found unchanged CRH and AVP mRNA levels in morphine dependent rats 7 days after morphine implantation. Furthermore, chronic morphine exposure by pellet implantation does not affect plasma PRL levels and dampens adrenocortical activation to stress challenges. In sharp contrast, chronic intermittent morphine administration results in elevated basal ACTH and CORT concentrations as well as facilitated pituitary-adrenocortical responses to novel stressors and is considered by itself as a chronic stressor [23].

It is intriguing that significant reductions in food intake and body weight at early phase of morphine treatment were not accompanied by overt changes in hypothalamic mRNA levels most of metabolic-related neuropeptides. Only NPY relative quantity values were moderately elevated in morphine treated- but not in pair fed rats. Decreased food intake may trigger only subtle changes of mRNAs that can not be technically detected over the substantial pool of neuropeptide mRNA in the hypothalamus.

At later time points during morphine exposure, tolerance develops in body weight gain and food consumption in morphine treated rats, still without any significant change in the expression of metabolic-related hypothalamic neuropeptides by the end (7^th^day) of the experiment. We have previously reported an elevation of NPY expression in hypothalamic samples of chronic morphine treated rats [16]. Furthermore, increased hypothalamic levels of NPY and co-expressed Agouti-related peptide (AgRP) mRNA were detected in mice chronically exposed to morphine [24]. By contrast, in the present study, we did not reveal significantly increased NPY mRNA levels on the final day (day 7^th^) that might be explained either by species difference or by different morphine treatment paradigm used in the present experiment.

Expression of orexigenic NPY, however, was elevated in pair fed animals 8 days after treatment. Fasting or food restriction and decreased leptin plasma levels are reliable stimuli for hypothalamic NPY transcription [25]–[27] and elevated NPY mRNA levels might be compensatory responses of pair fed animals. It is unknown however, why NPY transcripts remain unchanged in the hypothalamus of morphine treated animals which consume less food than placebo-treated, *ad libitum* fed rats and have lower circulating leptin level.

Recent microarray studies on various brain sites have revealed several genes that are differentially expressed in morphine-treated mice compared to controls [24], [28]. Within the mouse hypothalamus NPY and AgRP mRNA levels were upregulated while POMC mRNA down-regulated by 4 days morphine treatment [24]. In contrast, our study on rats did not detect significant changes in NPY and POMC mRNA levels in morphine dependent rats that might be due to species difference and differences in timing and dose of morphine.

It remains unrevealed to what extent these relative quantity values of neuropeptide mRNAs reflect alterations in peptide concentration and/or release. For instance, in morphine treated mice, hypothalamic NPY exhibited an increase in both mRNA and peptide levels, while CART and POMC/α-MSH exhibited slight down-regulation of mRNA levels with up-regulation of peptide levels [24]. mRNA stability and regulation by microRNAs might also contribute to the final regulation of hypothalamic neuropeptide expression in morphine dependent animals.

### Morphine Withdrawal

In the second part of the study, we have characterized stress- and metabolic-related physiological, hormonal and transcriptional events associated with acute morphine withdrawal.

We confirmed the finding that naloxone-precipitated morphine withdrawal results in significant activation of the hypothalamo-pituitary-adrenocortical axis [3], [29], here by measuring increased plasma levels of ACTH CORT and PRL. In contrast to transient pituitary hormone release seen during other acute stress situations [17], ACTH and CORT elevation during naloxone-precipitated morphine withdrawal lasts up to 2 hours after naloxone injection.

Early in progression of drug withdrawal, a rapid burst of hypothalamic CRH and UCN2 mRNA expression occurs that is paralleled with significant induction of stress hormones, withdrawal–induced weight loss and other physical symptoms of withdrawal. By contrast, relative quantity of POMC mRNA in the hypothalamic samples gradually decreased. NPY fold change values were only transiently elevated.

Using *in situ* hybridization histochemistry, we previously showed that activation of HPA axis during morphine withdrawal is accompanied by induction of *de novo* transcription of CRH gene in the medial parvocellular subdivision of the hypothalamic paraventricular nucleus [3] the cell population that initiates the neuroendocrine stress response. Our present qRT-PCR measurements also provided evidence for increased hypothalamic CRH mRNA levels in morphine dependent rats as early as 15 min after withdrawal that peaked 60 min after naloxone administration. Increase of hypothalamic CRH expression in morphine tolerant rats is specific to opioid withdrawal, since stress, caused by saline injection did not result in significant elevation of CRH mRNA relative quantities. In view of substantial pool of CRH peptide accumulated in the nerve endings parvocellular neurons at the median eminence and the rapidity of its release during morphine withdrawal [30] CRH mRNA increase described here seemed to be viewed most aptly as to serve to replenish depleted neuropeptide stores. The possible molecular mechanisms that drive CRH expression during morphine withdrawal may include activation of cAMP-pCREB pathway that has been shown upregulated in the tolerant rats’ PVH after naloxone injection [31].

CRH appears to be elevated during active withdrawal in many drugs of abuse [32]–[34]. Increased CRH expression in morphine treated, naloxone injected rats may also play a role in mediating metabolic and behavioural symptoms of opiate withdrawal. In addition to its well-known hypophyseotrophic effect, CRH is released centrally and mediates several stress-specific changes in food intake and energy metabolism through CRF-1 receptors [35], [36]. Central CRH inhibits food intake and increases sympathetic activity [37], [38] both effects contribute to metabolic changes seen in morphine dependent rats during withdrawal.

The weight loss during naloxone precipitated morphine withdrawal is very rapid and remarkable. Changes in body weight after spontaneous or naloxone precipitated morphine withdrawal are well documented [19], [39]. Here, the time course of weight loss has been revealed: as early as 15–30 min following naloxone injection, rats lost significant amount of weight, which can be due to summation of water loss associated with diarrhoea, salivation, lacrimation and rhynorrhea. Morphine withdrawal is accompanied with significant release of arginine vasopressin (AVP) into the blood [40] that might be interpreted not only as a rebound effect to tolerance developed after primary inhibition of AVP release by morphine but also as a response to dehydration (due to diarrhoea, salivation and lacrimation). To support this hypothesis we found a transient increase in hypothalamic AVP mRNA level 15 min after naloxone injection in morphine tolerant rats using RT-PCR. It remains unknown however, if increased AVP mRNA levels are localized in magnocellular neurons of the hypothalamic supraoptic (SON) and paraventricular nuclei (PVN) in morphine withdrawn rats. In a previous study increased neuronal activation has been detected in the neurohypophyseal neurons of the PVN and SON without any significant activation of AVP hnRNA levels in these cells two hours after morphine withdrawal in rats. Another important notion regarding AVP mRNA levels is the increase seen in placebo implanted naloxone injected rats implicating a tonic inhibitory effect by the endogenous opioids.

In support the role of CRH in withdrawal anxiety and anorexia it has been recently shown that CRF-1 receptor antagonists CP154526 and antalarmin reduced weight loss and irritability during naloxone precipitated morphine withdrawal in rats [9]. Functional anatomical results also revealed neuronal activation of autonomic-related hypothalamic neurons, such as those in the ventral parvocellular-, lateral and dorsal parvocellular subdivisions in the PVN [3], although it remains unknown if these autonomic projection neurons synthesize CRH in response to morphine withdrawal. Naloxone injection to morphine-dependent rats also increased c-Fos-IR in the central amygdala and bed nucleus of the stria terminalis, however these were CRH negative neurons [2].

The other noteworthy finding of the present experiments is to reveal induction of urocortin 2 expression in the hypothalamus of morphine dependent rats after naloxone injection. Urocortin 2 is a specific ligand of CRF-2 receptors [41], [42] and has been implicated in mediation of autonomic effects of stress [43], including anorexia [6]. To support this finding it has been revealed that CRF-2 receptor specific antagonist anti-sauvagine prevented the withdrawal-induced weight loss in chronically morphine treated rats [44]. Along these lines, mice with genetic disruption of CRF-2 receptor pathway display reduced somatic signs to stressful condition of spontaneous or naloxone-precipitated morphine withdrawal. However, analysis of weight loss in morphine dependent CRF2^−/−^ vs. WT mice did not reveal genotype or opiate treatment effect and genotype×morphine interaction [45].

Some of the efferent mechanisms related to opiate withdrawal-induced weight loss in rats may be associated with increased sympathetic outflow, initiated by neuropeptides of the CRF family [46]. It has been shown that autonomic related parvocellular neurons in the PVH- those which are activated during morphine withdrawal- are command neurons that regulate and coordinate sympathetic outflow [37]. Furthermore, central and peripheral CRH and UCN2 inhibit gastric emptying and promote colonic motility [47], [48] that may also contribute to gastrointestinal symptoms and weight loss during opioid withdrawal.

Post-synaptic mu opioid receptors (MOR) have been revealed in the hypothalamic arcuate nucleus [49] that are coupled to inhibitory G proteins and their activation hyperpolarize POMC neurons [50]. Furthermore, POMC expression in the hypothalamic arcuate nucleus is under negative autoregulation by opioids [51], [52]. In contrast, we and others did not detect significant downregulation of POMC mRNA levels in the hypothalamus of morphine treated rats [16]. If chronic morphine treatment inhibits POMC neurons, one can expect activation of POMC neurons following morphine withdrawal. However, Lightman and Young [22] and, more recently, Houshyar et al. [19] reported unchanged in situ hybridization signals corresponding to POMC mRNA over the arcuate nucleus of rats 4 h after naloxone precipitated- and 12 h–8 days following spontaneous morphine withdrawal, respectively. In contrast, we found significantly decreased POMC mRNA levels in the hypothalamus of morphine-implanted rats up to 2 h of naloxone injection. POMC expressing cells in the medial basal hypothalamus have two major posttranslational products, the endogenous opioid peptide β-endorphin and the anorexigenic alpha-melanocyte-stimulating hormone (αMSH). Due to translational and/or post-translational changes in neuropeptide expression however, it cannot be assumed that the hypothalamic peptide levels were changed along with mRNA content.

The molecular mechanisms responsible for morphine withdrawal-induced decline of POMC mRNA remain to be explored. In addition to yet unknown mechanisms mediated by opioid receptors, withdrawal-induced glucocorticoids may play a role in regulation of POMC mRNA levels either by decrease RNA stability and/or enhance its degradation since the short time frame of POMC mRNA down-regulation is inconsistent with the classic genomic effect of activated glucocorticoid receptors. It has been shown that GR complexes can be bound to untranslated 3′ end of target mRNA to facilitate its degradation [53]. Furthermore, rapid decay of POMC mRNA levels in the hypothalamus is compatible with activation of various microRNA (miRNA) populations. To support this hypothesis, a search of miRNA databases (www.targetscan.org, www.microrna.org) revealed several miRNA that might interact with POMC mRNA untranslated region, including miR-488, miR-485, miR-384-3p, miR-383, miR-377, miR-485-5p and miR-181 (family).

The other major metabolic related cell group in the medial basal hypothalamus is the NPY containing neuron population in the arcuate nucleus. We found transient upregulation of NPY mRNA levels in the hypothalamus 30 min after naloxone injection to morphine dependent rats, although it remains unknown how this increased mRNA is translated as increased NPY release at various hypothalamic targets. Because attenuated withdrawal syndrome is seen after intracerebroventricular application of NPY [54], [55] and because NPY is an orexigenic peptide, it can be hypothesized that NPY is involved in an endogenous counter-regulatory mechanism to opioid withdrawal.

In conclusion, this study shows differential transcriptional responses of selected stress- and metabolic-related genes in the hypothalamus of morphine dependent rats during naloxone precipitated withdrawal. Thus, drug abstinence triggers corticotropin-releasing hormone and urocortin 2 mRNA levels while dampens proopiomelanocortin mRNA relative quantities in hypothalamic samples. These transcriptional changes might be related to the regulatory/counter regulatory mechanisms in response to acute drug withdrawal and/or to replenish depleted neuropeptide stores in hypothalamic neurosecretory neurons.
